# Estimating female malaria mosquito age by quantifying Y-linked genes in stored male spermatozoa

**DOI:** 10.1038/s41598-022-15021-z

**Published:** 2022-06-22

**Authors:** Damian Madan, Rafael Rivera, Corrie Ortega, Justin C. Touchon, Corinna Kimball, Geert-Jan van Gemert, Wouter Graumans, Stephanie Matsuura, Sean S. Parghi, David Bell, Teun Bousema, Chris Drakeley, Katharine A. Collins, Thomas R. Burkot

**Affiliations:** 1 Global Health Labs, Bellevue, WA 98007 USA; 2 Intellectual Ventures Global Good Fund, Bellevue, WA 98007 USA; 3 Vassar College, Poughkeepsie, NY USA; 4 Department of Medical Microbiology, Radbound University Nimegen Medical Centre, Nimegen, The Netherlands; 5 Department of Infection Biology, School of Hygiene and Tropical Medicine, London, UK; 6 Australian Institute of Tropical Health and Medicine, James Cook University, Cairns, QLD Australia; 7 Present Address: Anavasi Diagnostics, Seattle, WA 98195 USA; 8 Present Address: Ozette, Seattle, WA 98195 USA; 9 Present Address: School of Medicine, University of Hawaii, Honolulu, HI 96813 USA; 10 Present Address: Gilead Sciences, Inc, Seattle, WA 98102 USA; 11 Present Address: Independent Consultant, Issaquah, WA 98027 USA

**Keywords:** Parasitology, Microbiology techniques

## Abstract

Vector control strategies are among the most effective measures to combat mosquito-borne diseases, such as malaria. These strategies work by altering the mosquito age structure through increased mortality of the older female mosquitoes that transmit pathogens. However, methods to monitor changes to mosquito age structure are currently inadequate for programmatic implementation. Female mosquitoes generally mate a single time soon after emergence and draw down spermatozoa reserves with each oviposition cycle. Here, we demonstrate that measuring spermatozoa quantity in female *Anopheles* mosquitoes is an effective approach to assess mosquito age. Using multiplexed qPCR targeted at male spermatozoa, we show that Y-linked genes in female mosquitoes are exclusively found in the spermatheca, the organ that houses spermatozoa, and the quantity of these gene sequences significantly declines with age. The method can accurately identify mosquitoes more than 10 days old and thus old enough to potentially transmit pathogens harbored in the salivary glands during blood feeding. Furthermore, mosquito populations that differ by 10% in daily survivorship have a high likelihood of being distinguished using modest sample sizes, making this approach scalable for assessing the efficacy of vector intervention control programs.

## Introduction

The intensity of transmission of mosquito-borne pathogens including malaria is a function of vulnerability and receptivity where vulnerability is a measure of the incidence of the pathogen in the human population while receptivity estimates the capacity of the environment to support vector populations. Receptivity is a function of adult mosquito abundance, which depends on the capacity of larval habitats to produce adult mosquitoes, the quantity of blood meal hosts available, the survivorship or lifespan of the mosquito, and the vector control strategies deployed. Adult vector control is central to most strategies to mitigate malaria and other mosquito-borne diseases. For instance, insecticide treated nets and indoor residual spraying were estimated to be responsible for 80% of the reduction in *P. falciparum* malaria in Africa^1^.

Evaluating changes in malaria transmission intensity as a result of vector control can be measured in the vectors directly by monitoring the entomological inoculation rate (EIR) or indirectly by evaluating the components of vectorial capacity in mosquitoes. However, the available vector surveillance tools to measure these parameters have limited sensitivity or are constrained by the sampling effort required^2^. While immunological and molecular methods can quantify changes in receptivity and transmission by monitoring sporozoite rates and EIRs^3–5^, use of these tools are limited to high transmission areas^3^. In areas of low transmission, as the abundance of the vector population decreases, the sampling effort required to estimate the sporozoite rate or EIR become increasingly more challenging as ever larger numbers of mosquitoes are required to accurately determine the sporozoite rate^6^. In low transmission scenarios approaching or following malaria elimination, the options for monitoring transmission potential are thus limited to monitoring vector abundance or vector survivorship^6^. Vector abundance, however, is notorious for its heterogeneity, varying widely in both time and space and being unpredictable across ecological settings^7^. While infection rates in mosquito populations are heavily affected by mosquito age structure, mosquito age is less affected by the bias introduced by environmental and sampling noise relative to infection data^8^.

Methods for estimating mosquito survivorship include ovarian dilatation counts^9^, near^10,11^ and mid^12^ infra-red scanning (NIR and MIR, respectively) profiles, gene transcript abundance^13^, mark-recapture experiments, and comparisons of sporozoite and oocyst infection rates^14^. Counts of dilatations on ovary stalks provide epidemiological relevant estimates of the proportion of mosquitoes in an area that have lived long enough to transmit malaria (e.g., dilatations represent individual ovipositions that a female mosquito has made with ovipositions generally occurring every 3–5 days)^9^. This technique is sensitive in allowing the age structure of a vector population to be defined in increments equal to the length of the oviposition cycle (largely equivalent to the frequency of blood feeding). However, the technique is laborious, requires live mosquitoes, and is technically challenging to perform. Parity rates divide female populations into two classes: nulliparous (those that have not yet oviposited) and those that have laid eggs one or more times. While easier to perform than ovarian dilatation dissections, parity rates still require live specimens and generate insensitive binary estimates of the age of a population (i.e., less than 2–3 days after emergence or > 2–3 days).

NIR and MIR scanning use absorbance profiles generated from live or dead mosquitoes to estimate mosquito ages. However, the age estimates from NIR have wide confidence intervals, as NIR profiles are affected by the larval habitat from which the adults emerged, their diet, and their physiological state^15^. Thus far, NIR has only been evaluated with laboratory reared colony mosquitoes and a limited number of wild mosquitoes^10,11,15^. Recently, a deep learning MIR technique (DL-MIRS) was shown to have the potential to accurately predict the age of *Anopheles* into one of three age categories with limited sampling. This method was validated by comparing MIR scanned mosquitoes to their age as determined by the ovarian dilatation technique^16^. Further evaluation against other age grading techniques is needed to validate the DL-MIRS. Adult mosquito ages estimated by mosquito gene transcript abundance may, like NIR, be affected by the larval habitat from which the adults emerged^13^.

Mark-recapture data and comparisons of oocyst and sporozoite infection rate data are infrequently used to estimate age structure due to the intensive labor requirements and associated costs^6^. While capable of generating estimates of survivorship per feeding cycle and over the length of the extrinsic incubation period^7,14^, the challenges of collecting adequate numbers of infected vectors in low transmission areas limits where infection rate data can estimate survivorship. Hence, a critical need remains for an age-grading method that can monitor changes in population age to monitor both receptivity and the impact of vector control interventions.

Female mosquitoes, with few exceptions, mate a single time shortly after emergence^17–19^ and store several thousand spermatozoa in spermatheca^20^. Following bloodmeals at 2-to-4-day intervals, eggs develop and are fertilized just before oviposition. Hence, spermatozoa numbers incrementally diminish with each oviposition at intervals equal to the length of the oviposition/feeding cycle and by 1–10 spermatozoa per egg laid^21^. This aspect of the biology of the female mosquito presents an opportunity to estimate mosquito ages as spermatozoa quantity will decrease with age. Male spermatozoa, in particular, pose an intriguing target to measure the effect, as these cells harbor the only Y chromosomes within the female mosquito.

Here, female mosquito ages were assessed by quantifying spermatozoa using qPCR targeted to genes found exclusively on Y chromosomes. We show a strong negative correlation between Y-linked gene copy numbers and mosquito age, and data simulations based on these measurements suggest that this method could distinguish age structure differences among mosquito populations using manageable sample sizes. Together these data demonstrate the potential of quantitative measures of spermatozoan counts for determining epidemiologically relevant ages of the malaria vector, *Anopheles stephensi*.

## Results

We designed a qPCR assay to measure sequences found exclusively on *A. stephensi* Y chromosomes. The reaction targeted both the single copy *GUY1* and multicopy *YG2* Y-linked genes^22,23^ and *RPS*6, which served as an autosomal control (Supplemental Fig. 1).

**Figure 1:**
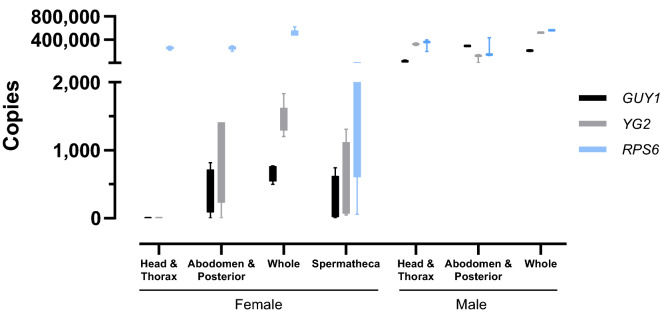
Y-linked sequences are associated with spermatozoa in female mosquitoes. Copy numbers of Y-linked and autosomal gene sequences in sectioned female and male mosquitoes were measured using qPCR.

When we dissected 2–6 day old female mosquitoes to obtain fractions with and without spermatheca, *RPS6* amplification was robust in all sections and roughly scaled with the fraction of the mosquito added to the reaction (Fig. 1). Only sections containing spermatheca, including the spermatheca alone, resulted in amplification of *GUY1* and *YG2*. In male mosquitoes, where Y-linked sequences are expected to be present in all DNA-containing cells, *GUY1* and *YG2* copy numbers were similar in magnitude to *RPS6*. Together these results strongly suggest that amplification of *GUY1* and *YG2* in female mosquitoes coincides with the presence of male spermatozoa.

**Figure 2:**
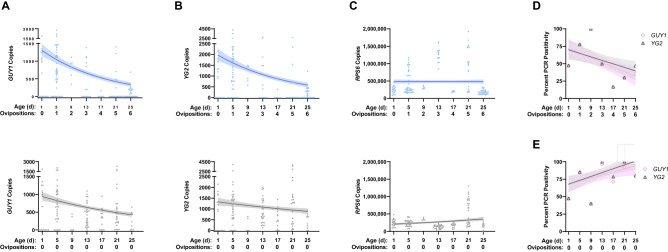
Y-linked gene copies significantly decrease with age. Copy numbers of *GUY1* (**A**), *YG2* (**B**), and *RPS6* (**C**) were measured in mosquitoes differing in age from blood-fed (top) and blood-denied (bottom) cohorts prepared using the manual method. Fits (solid lines) with 95% confidence intervals (shaded regions) from zero-inflated negative binomial GLM are displayed. The percent of PCR positive measurements as a function of age for Y-linked sequences and linear regression fits (solid lines) with 95% confidence intervals (shaded regions) are displayed for blood-fed (**D**) and blood-denied (**E**) cohorts.

We applied the method to mosquitoes that emerged simultaneously and were then immediately divided into two cohorts. One cohort was denied access to blood and thus could not lay eggs. The second cohort was blood fed and oviposited at 4-day intervals (Fig. 2). Measurements of gene copies best fit a negative binomial distribution. Given the prevalence of zeros in the dataset, we used zero-inflated generalized linear models (GLM) with a negative binomial distribution to explicitly test for differences between the number of gene copies present in blood-fed and blood-denied mosquitoes. In all models, mosquito age and blood feeding treatment and their interaction were independent variables, and the number of gene copies was the dependent variable. There was a statistically significant interaction between feeding treatment and age for both *GUY1* (χ^2^ = 20.10, *P* < 0.0001) and *YG2* (χ^2^ = 25.49, *P* < 0.0001) indicating that while copy numbers generally decreased with age, the slope of the decrease differed between blood-fed and blood-denied mosquitoes (Fig. 2). Indeed, the slope of the decrease for *YG2* in blood-denied mosquitoes was not different from zero. Statistical results were nearly identical when zeros were excluded and data were analyzed with negative binomial GLMs (results not shown).

As expected, the autosomal control *RPS6* copy number showed no obvious dependence on mosquito age (Fig. 2). We analyzed blood-fed and denied mosquitoes separately and revealed no relationship between age and copies of *RPS6* for blood-fed mosquitoes (χ^2^ < 0.001, *P* = 0.99) but a positive relationship for the blood-denied cohort (χ^2^ = 9.43, *P* = 0.002), which may be an artifact of primer concentrations biasing Y-linked gene targets in the multiplexed reaction (Supplemental Table 1).

While qPCR is a versatile and widespread technique, it requires expensive equipment and precise execution. End-point PCR and isothermal nucleic acid amplification technologies are methods that may be more suitable for some resource-limited situations^24^. To ascertain whether end-point measurements of Y-linked genes could be used to quantify differences in mosquito age structure, we reanalyzed the data using qPCR positivity as a surrogate for end-point PCR positivity. A linear regression model using qPCR positivity and age as independent variables found *GUY1* (*R*^2^ = 0.09334, *P* = 0.0003) and *YG2* (*R*^2^ = 0.05375, *P* = 0.006) were significantly influenced by mosquito age for blood-fed mosquitoes (Fig. 2D), and a reverse or absent effect was seen with blood-denied mosquitoes (Fig. 2E; *GUY1*: *R*^2^ = 0.04473, *P* = 0.0135; *YG2*: *R*^2^ = 0.08164, *P* = 0.0007).

To attempt to overcome potential issues associated with differing sample preparation efficiencies, we combined data from *GUY1*, *YG2*, *RPS6*, and *KLH*, a gene not found in mosquitoes that was added prior to sample preparation as a process control. Combining Y-linked measurements and normalizing to *RPS6* or *KLH* did not substantially affect results (results not shown and Supplemental Fig. 2), which again may be a result of primer biasing.

**Figure 3:**
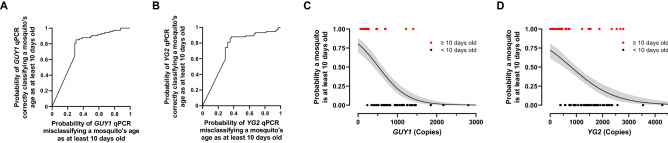
Probabilities of determining if mosquitoes are old enough to pose health risk. ROC analysis was performed on *GUY1 *(**A**) and *YG2 *(**B**) copy numbers for mosquitoes younger and older than 10 days. Data for *GUY1* (**C**) and *YG2* (**D**) were also analyzed excluding zeros using a binomial GLM. Fits (solid lines) with 95% confidence intervals (shaded regions) are displayed. Raw gene copy number values (circles) for the data sets used to generate the fits are shown for display purposes.

Prior to 10 days of age, mosquitoes are generally incapable of harboring *Plasmodium* sporozoites, and thus do not pose a malaria health risk^25^. We performed receiver operator characteristic (ROC) analysis to determine how well qPCR of Y-linked genes could predict whether a mosquito is older than 10 days and thus potentially infectious to humans. Areas under the curve (AUC) for *GUY1* and *YG2* were 0.7520 and 0.7264, respectively (Fig. 3A,B). We also performed a binomial GLM on the same data excluding zeros, which demonstrated that as gene copy number decreases, the probability that a mosquito is older than 10 days old increases for both *GUY1* and *YG2* (Fig. 3C,D).

Previously a semi-high-throughput (semi-HTP) method was developed to improve upon the labor-intensive protocol for determining the malaria infection status of mosquitoes^26^. In this semi-HTP method mosquitoes and silica beads are placed within individual wells in 96-deepwell plates, and homogenization is performed by vigorous mechanical plate shaking or bead beating.

In the present study, the manual method used to digest mosquitoes prior to qPCR is labor intensive. We sought to improve the ease of mosquito digestion by employing the semi-HTP protocol prior to qPCR. The semi-HTP method was unmodified from the previous report^26^. As *KLH* normalization imparted modest changes to the significance of the effects of the previous experiment (Supplemental Fig. 2), it was excluded from this experiment.

Once again, we observed that copy numbers significantly decreased with age for both *GUY1* (χ^2^ = 30.75, *P* < 0.0001) and *YG2* (χ^2^ = 37.16, *P* < 0.0001) (Supplemental Fig. 3). This decrease was more pronounced for blood-fed than denied mosquitoes, but considerably less so than for samples prepared with the manual method, as noted by nearly significant or marginally significant interactions between feeding treatment and age (*GUY1*: χ^2^ = 5.43, *P* = 0.066; *YG2*: χ^2^ = 5.83, *P* = 0.054). Copy numbers of RPS6 increased slightly with age (age: χ^2^ = 5.68, *P* = 0.017), an effect which was more pronounced in blood-denied than fed mosquitos (interaction: age: χ^2^ = 11.65, *P* = 0.0006) and may again be an artifact of primer biasing (Supplemental Table 1).

The absence of qPCR signals was considerably more common using the semi-HTP method, leading to nonsignificant trends when PCR negativity samples were excluded for either *GUY1* (age: χ^2^ = 1.13, *P* = 0.29, blood: χ^2^ = 1.72, *P* = 0.19, interaction: χ^2^ = 1.25, *P* = 0.26) and *YG2* (age: χ^2^ = 0.37, *P* = 0.54, blood: χ^2^ = 3.10, *P* = 0.078, interaction: χ^2^ = 1.82, *P* = 0.18) (Supplemental Fig. 3). These effects may be due to insufficient liberation of spermatozoa DNA. The semi-HTP protocol was unchanged from previous studies and employed relatively short bead beating and proteinase K digestion times^26^. In optimizing the manual method, we found increasing the duration of these steps led to decreased variability and qPCR negativity (Supplemental Fig. 4).

**Figure 4:**
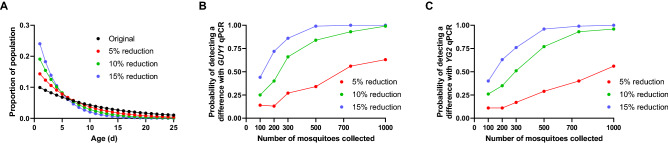
Probabilities of differentiating groups with differing age structures before and after intervention. Groups of mosquitoes with different age structures (**A**; colored lines) were simulated to quantify the ability to detect differences using qPCR targeting *GUY1* (**B**) or *YG2* (**C**) copy numbers. The probability of detecting a statistically significant difference between the original population and one of the post-intervention groups was measured using a zero-inflated GLM assuming a negative binomial distribution.

We next sought to understand how useful our approach could be in determining a significant difference in the number of Y-linked gene copies present in two cohorts of mosquitos, such as might occur after a vector control intervention deployment. As a successful intervention that increased mosquito mortality would shift the median age of the population younger, a greater number of Y-linked gene copies would be present in the post-intervention cohort. To estimate a range of possible scenarios, we simulated three different interventions that reduced the daily survival rate by 5, 10, and 15%, respectively (Fig. 4A). We then estimated the ability to detect a statistical difference between the pre-intervention population (with a daily survival rate of 91%) and our simulated intervention populations assuming six different sample sizes ranging from 100 to 1000 mosquitos in each sample. We separately simulated the distribution of gene copies of *GUY*1 and *YG*2.

As might be expected, increasing both the efficacy of an intervention and the sample size dramatically improve the ability to detect a difference between two cohorts (Fig. 4B,C). A 5% reduction in daily survival corresponded to an estimated reduction in the mean age of the population of 1.9 days, whereas a 15% reduction in daily survival shifted mean population age by 4.4 days. Viewed from the perspective of the probability of detecting differences in the age structure of mosquito populations, populations that differ by 10% in the daily mortality rate can reasonably be detected by changes in gene copy numbers in a sample of only 500 mosquitoes (Fig. 4B). Even a weakly successful intervention (e.g., 5% reduction in daily survival) has a 63% likelihood of being detected with a sample of 1000 mosquitos collected before and after intervention. *GUY*1 provided a slightly greater ability to detect a difference than simulations based on *YG*2 (Fig. 4C).

## Discussion

Here we show that as female mosquitoes age, copy numbers of Y-linked genes significantly decline. We observed the greatest effect in groups of mosquitoes that oviposited, supporting the hypothesis that Y-linked genes are exclusively associated with spermatozoa in female mosquitoes and that the quantity of these genes declines as fertilized eggs are laid. Y-linked gene copy numbers also decreased for mosquitoes denied blood meals and thus are incapable of ovipositing, suggesting that other effects, such as sperm death, may also contribute to Y-linked gene copy numbers declining over time.

When considering options for quantifying spermatozoa, we selected methods that could be used to estimate mosquito population age distributions in malaria elimination scenarios. PCR-based approaches are appealing because a range of platforms exist, including those that offer high degrees of automation^24^. Indeed, multiplexed qPCR has been used on large numbers of captured mosquitoes for dengue virus control, including ascertaining *Wolbachia-*infections^27^.

For Y-linked sequences we targeted both the single copy *GUY1* and multicopy *YG2* genes. We surmised that the greater number of *YG2* sequences would decrease data variability. However, *YG2* results mostly mirrored those of *GUY1*, albeit at approximately a threefold greater quantity, indicating the sequences are linked closely enough that the sample preparation methodologies greatly favor all or none of the sequences being present in the reaction. The loci of these sequences remain mostly unknown, as mosquito Y chromosomes are notoriously difficult to sequence due to high degree of repetition^23^. Monitoring the ratio of *YG2* to *GUY1* copy numbers may serve as a useful quality control measure in populations where Y chromosome instability is suspected.

The manual sample preparation method we describe requires several manipulations. The semi-HTP method removes many hurdles to operationalizing sample preparation. However, the semi-HTP method was associated with greater variability and higher levels of qPCR negativity, especially in younger mosquitoes, an effect likely due to insufficient liberation of spermatozoa DNA from all samples. Future studies may benefit from optimizing digestion parameters.

Interestingly, even when extreme digestion was used in the manual method, some nulliparous mosquitoes still showed PCR negativity for Y-linked genes. It is possible that these mosquitoes were virgins or inseminated by sterile males. Further studies are needed to ascertain the cause of the effect and whether it is seen to the same extent in wild populations. Nevertheless, we found strong significant correlations between PCR positivity for Y-linked genes and mosquito age for both preparation methods. These results suggest that end-point analyses, such as PCR and isothermal nucleic acid amplification tests, could be used in the absence of qPCR availability.

Our data simulations suggest the manual method could be employed using manageable numbers of mosquitoes to distinguish age structure differences. Attractive targeted sugar baits were estimated to increase the daily mortality rate by 9%^28^. Using the technique described here, this change in age structure would likely be detectable in samples of 500 mosquitoes.

More work is needed to build on the overarching results of this proof-of-principle study to determine if the age of wild mosquitoes can be estimated by quantifying spermatozoa. This work could encompass studies on the natural biological variability inherent in mosquito populations, including Y-linked gene copy number stability within wild populations of *A. stephensi* and other mosquito species and strains and how spermatozoa counts in females are impacted by rearing temperature and male age at insemination. Further potential application of this approach to age grading might refine spermatozoa quantitation by investigating alternative sample preparation methods, including pooling of samples, improved normalization strategies, and quantitative measures, such as digital droplet PCR. The assay also requires evaluation by comparing it against an established age-grading technique in field.

The reliability of estimating the age of female mosquitoes obtained by this novel approach will be a function of the variances in initial numbers of spermatozoa transferred during mating, the numbers of eggs laid per oviposition, the length of the oviposition cycle, and the numbers of spermatozoan lost per fertilized egg. Of these parameters, little is presently known in wild mosquito populations, but this tool provides a means to quantify these important aspects of the biology of mosquitoes.

Community based vector control, such as insecticide-treated bed nets (ITN) and indoor residual spraying (IRS) significantly reduce vector numbers and the proportion of vectors that are infectious. Eliminating malaria will require additional interventions to act synergistically with ITNs and IRS. Estimates of the impact of any additional interventions on the vector population will be difficult to monitor by changes in population numbers or vector infection rates. Hence, there is a critical need for a sensitive method to monitor changes in the age structure of the vector population and, in particular, that portion of the population that is sufficiently old to be infected with the infectious sporozoite stage of the malaria parasite. Here, we show a proof of principal for a novel age-grading approach based on monitoring the decrease in male spermatozoan counts in female mosquitoes, a potentially powerful tool to evaluate vector control strategies that can help control and eliminate malaria.

## Materials and methods

### Reagents

Primers and probes were purchased from IDT (Coralville, IA, USA) and from LGC, Biosearch Technologies (Petaluma, CA, USA). *GUY1* and *RPS6* linear fragments were purchased from GeneWiz (South Plainfield, NJ, USA). *YG2* linear fragment and *KLH07* plasmid were purchased from IDT (Coralville, IA, USA). EZ1 Advance XL and EZ1 DNA Tissue Kits were purchased from Qiagen (Hilden, Germany). TaqMan™ Fast Advanced Master Mix, 10 mM dNTP Mix, 25 mM MgCl_2_, and DEPC-Treated Water were purchased from ThermoFisher Scientific (Waltham, MA, USA). KAPA3G Plant PCR Kit was purchased from Roche Sequencing and Life Science (Indianapolis, IN, USA). ZR BashingBead (2 mm) Lysis Tubes was purchased from Zymo Research (Irvine, CA, USA). Mini-Beadbeater-16 was purchased from BioSpec Product, Inc. (Bartlesville, OK, USA). Proteinase K originated from the Qiagen (Hilden, Germany) EZ1 DNA Tissue kit.

### Mosquito preparation

#### Manual method

*Anopheles stephensi* clone STE 2, originally from India, was obtained from the Malaria Research and Reference Reagent Resource Center (MR4) and reared at The Center for Global Infectious Disease Research (Seattle, WA) as previously described^29^. One day after emergence, mosquitoes were transferred to Global Health Laboratories (Bellevue, WA) and randomly separated into blood-fed and blood-denied cohorts in separate cages. Mosquitoes were allowed to feed on arms of human volunteers or Lillie glass feeders containing fresh human blood collected in Vacutainer^®^ PST™ Tubes (BD, Oakville, ON)^29^. Female mosquitoes that had not visibly engorged were immediately removed and discarded. After each oviposition and immediately prior to subsequent blood feeding, approximately 20 mosquitoes from both cohorts were removed and stored at – 80 °C until further use.

Whole or dissected mosquitoes were individually placed in ZR BashingBead Lysis Tubes with eighteen 2 mm ceramic beads and 237.5 Buffer G2 from EZ1 DNA Tissue Kit. Tubes were placed in a Mini-Beadbeater-16 and, unless otherwise indicated, shaken for three 5 min intervals. Samples were incubated at 56 °C for 15 min to allow foam to dissipate. Then 12.5 μL of proteinase K was added and, unless otherwise indicated, samples were incubated at 56 °C for 20–24 h. Samples were vortexed, transferred to fresh microfuge tubes, homogenized by pipetting up and down 5–10 times, centrifuged at 10,000 rcf for 3 min, and transferred to 2 mL tubes provided by the EZ1 Tissue Kit. Unless otherwise indicated, 1 μL of 1000 copies/μL KLH07 linear fragment (IDT, Coralville, IQ, USA) was added to each sample, and DNA was extracted according to manufacturer’s recommendations for EZ1 Tissue Kit.

#### Semi HTP method

*Anopheles stephensi* (Sind-Kasur strain from Pakistan) mosquitoes were kept at 26 °C and 70–80% humidity, with a 12 h reverse day/night cycle at Radbound University (Nimegen, The Netherlands). One day after emergence, female mosquitoes were randomly separated into blood-fed and non-blood fed cages. Blood-fed mosquitoes were allowed to feed on membrane covered glass feeders containing fresh human blood collected in Lithium heparin Vacutainers every 5 days, and non-engorged mosquitoes removed after blood feeding. After the 1st, 3rd, and 5th oviposition and immediately prior to subsequent blood feeding, approximately 96 mosquitoes were collected from the blood fed cage. Similar numbers of mosquitoes were concurrently collected from the non-blood fed cage. Mosquitoes were stored in 15 mL Falcon tubes with silica gel beads at − 20 °C until processing. Mosquitoes were added to 96 well plates and homogenized by bead beating as previously described^26^. After homogenization 400 µL PBS was added, for a total volume of 500 µL per well. Plates were centrifuged at 2000 rpm for 5 min, and the supernatant transferred to new 96-deepwell plates. DNA was extracted on an automated MagNA Pure 96 system with a total NA large volume kit (Roche, Basel, CH), and eluted in a volume of 50 µL. Samples were directly frozen in plates at − 20 °C, shipped on dry ice to Global Health Laboratories (Bellevue, WA), then stored at − 80 °C until PCR.

### qPCR

For each reaction 5 μL of sample was added to a final volume of 25 μL, which included primers and probes (Supplemental Table 1) and, unless otherwise indicated, 2.5 mM MgCl_2_, 200 μM dNTP mix, and KAPA3G polymerase (0.16 U/μL). Reactions using AmpliTaq Fast DNA polymerase were run at 1X. Plates were sealed, vortexed for 20 s, pulse centrifuged, and run on a CFX96 (BioRad, Hercules, CA, USA) programed to run 95 °C for 5 min followed by 45 cycles at 95 °C for 15 s and 62.5 °C for 30 s. Final concentrations were extrapolated using manufacturer’s software from reaction wells containing seven-point standard curves (1.28–2^5^ c/µL).

### Statistical analyses

All data were analyzed in either Prism v9.2.0 (GraphPad Software Inc., La Jolla, CA) or R v4.1.0^30^. We analyzed the relationship between the number of gene copies present in blood-fed and blood-denied mosquitoes with zero-inflated generalized linear models (GLM) with a negative binomial distribution using the R package glmmTMB^31^. Mosquito age and blood feeding treatment and their interaction were independent variables, and the number of gene copies was the dependent variable. We analyzed each gene of interest (*GUY1*, *YG2*, or *RPS6*) and preparation method (Manual or Semi-HTP) separately. Final sample sizes were as follows. *GUY1*: Manual Fed (N = 139), Manual Denied (N = 136), Semi-HTP Fed (N = 343) Semi-HTP Denied (N = 415). *YG2*: Manual Fed (N = 139), Manual Denied (N = 136), Semi-HTP Fed (N = 338) Semi-HTP Denied (N = 409). *RPS6*: Manual Fed (N = 139), Manual Denied (N = 139), Semi-HTP Fed (N = 401) Semi-HTP Denied (N = 345). We compared slopes of the regressions between gene copies and age for blood fed vs denied mosquitos using the emmeans package^32^.

### Simulating the effect of an intervention

To estimate the likelihood of detecting an effect of an intervention on mosquito age, we simulated groups of mosquitos with different mean gene copies of both *YG2* and *GUY1*. Our underlying assumption was that a successful intervention would yield a population of younger mosquitos, which would therefore have an increased number of Y-gene copies present in females because they had had fewer opportunities to oviposit eggs. First, we used the parameter estimates and standard errors from a zero-inflated negative binomial GLM to estimate the relationship between age and gene copies in our lab-based experimental data. Models were run using the glmmTMB package in R^31^. We then simulated populations of mosquitoes of different ages based on a daily survival rate of 0.91, based on data from *A. gambiae*^28^. Once we had a population of mosquitoes of different ages, gene copies were assigned to each individual based on the model parameters. The number of gene copies per individual were simulated in a two-step process. First, based on our lab data we assigned a binomial probability of 0.40 to the number of gene copies being zero or not (i.e. there was a 40% chance that an individual had zero gene copies). If an individual did not have zero gene copies, the number of gene copies was randomly selected from a negative binomial distribution based on the age of the mosquito. In addition to simulating three levels of intervention success, we simulated populations of 100, 200, 300, 500, 750 and 1000 mosquitos in order to understand the impact of sample size on the ability to detect a difference in two cohorts of mosquitos.

## Supplementary Information

Supplementary Information 1.

Supplementary Information 2.

Supplementary Information 3.

Supplementary Information 4.

Supplementary Information 5.

Supplementary Information 6.

## Data Availability

The authors confirm that the data supporting the findings of this study are available within the article and its supplementary materials.
