# Supplementary figures and images for: Estimating female malaria mosquito age by quantifying Y-linked genes in stored male spermatozoa
Source: Sci Rep. 2022 Jun 22;12:10570. doi: 10.1038/s41598-022-15021-z (PMC9217924; doi:10.1038/s41598-022-15021-z)

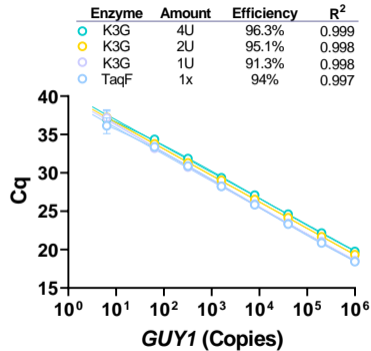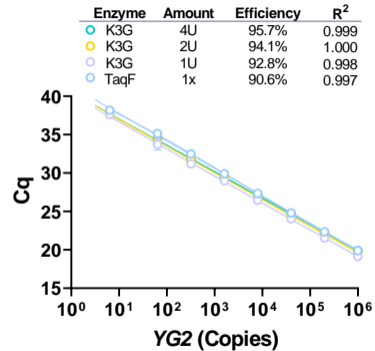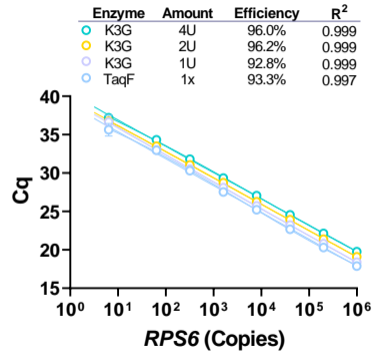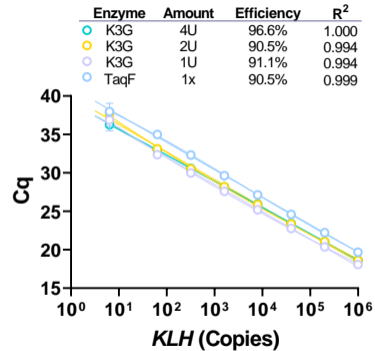

Supplement: Supplementary file 1 — Supplementary Information 1. [file 41598_2022_15021_MOESM1_ESM.pdf]

A

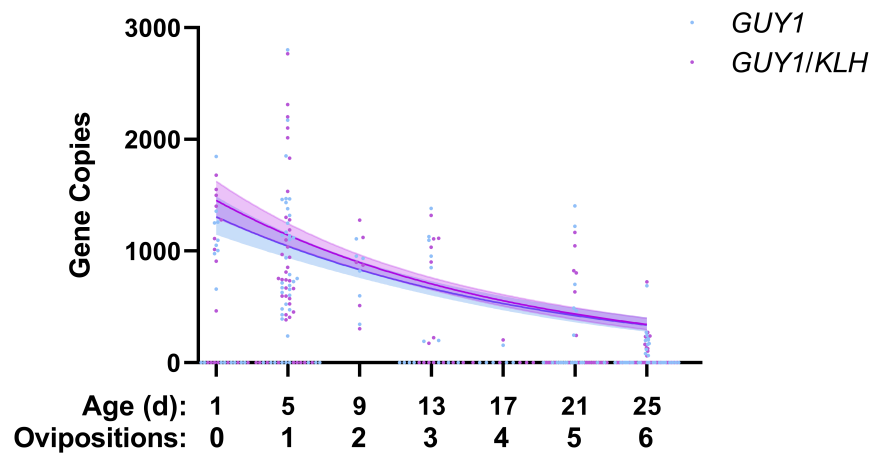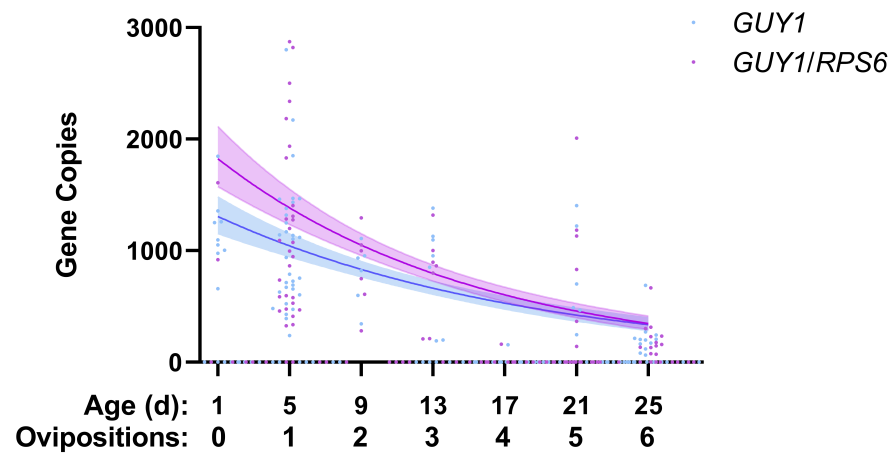

B

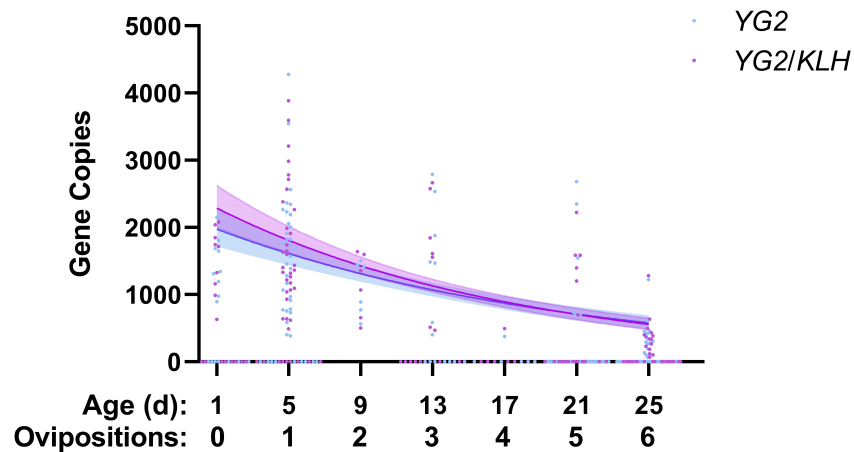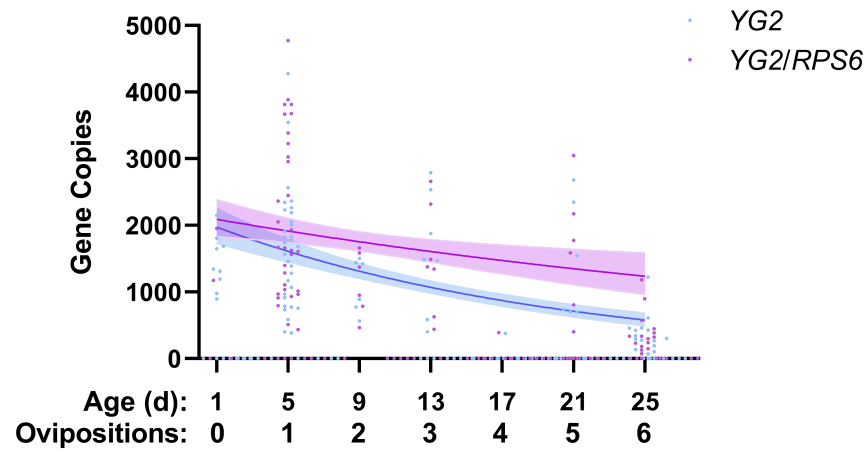

Supplement: Supplementary file 2 — Supplementary Information 2. [file 41598_2022_15021_MOESM2_ESM.pdf]

**A**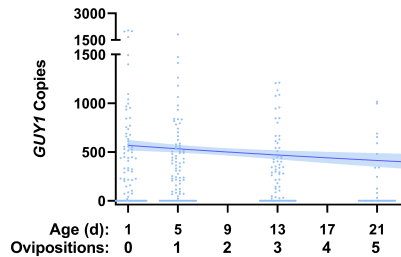**B**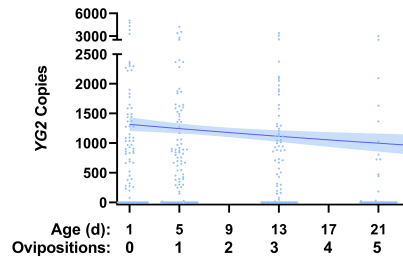**C**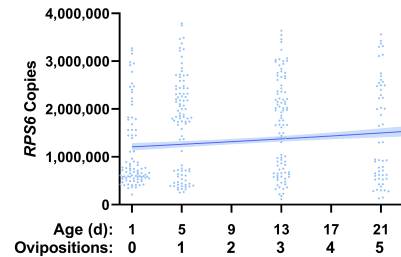**D**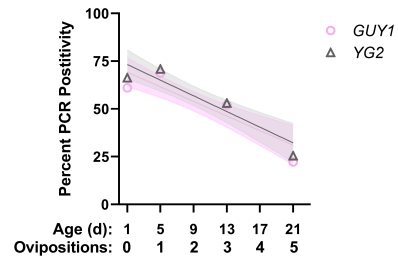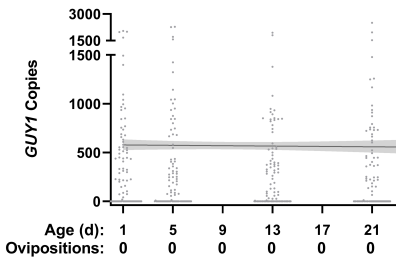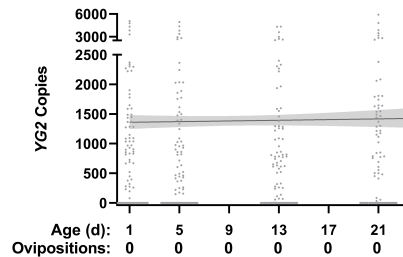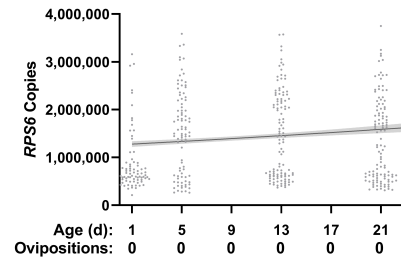**E**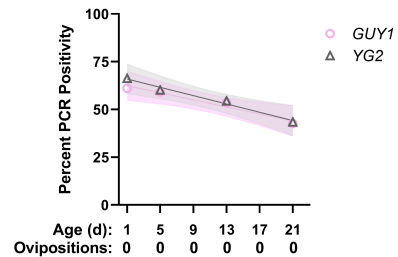

Supplement: Supplementary file 3 — Supplementary Information 3. [file 41598_2022_15021_MOESM3_ESM.pdf]

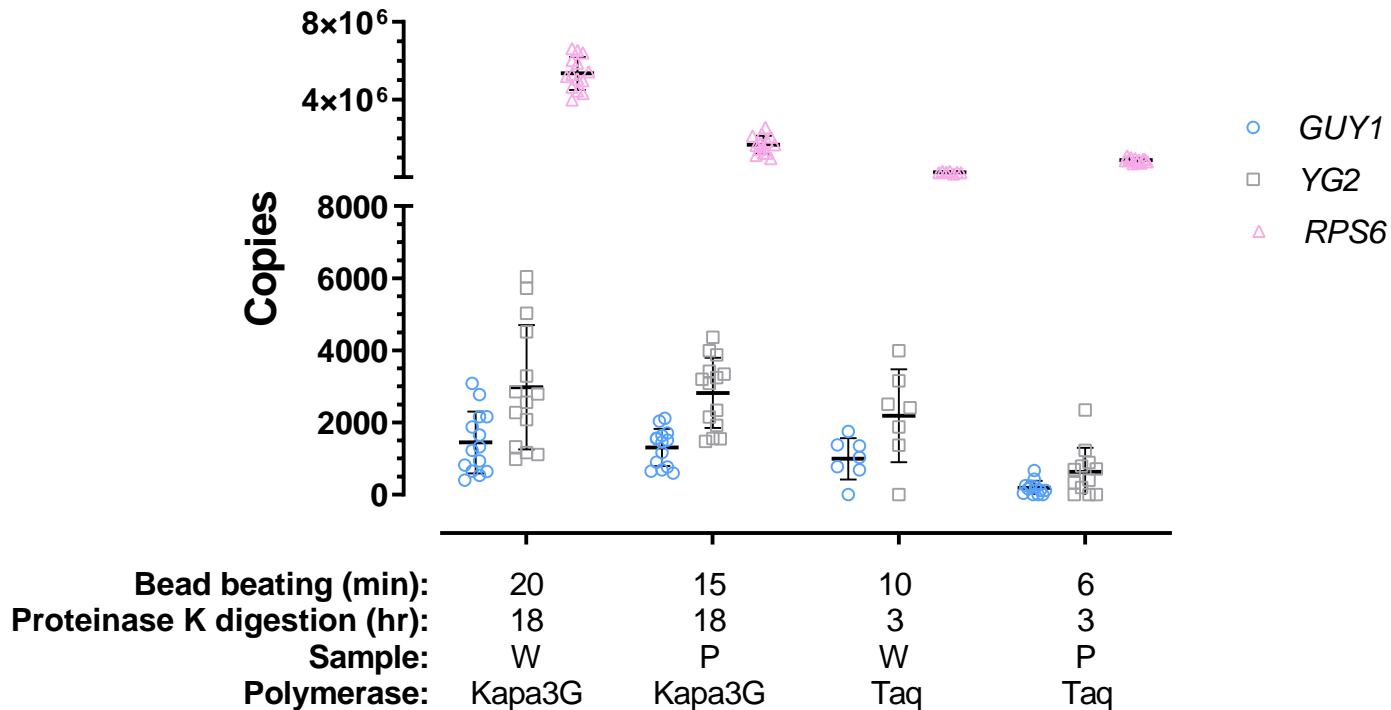

Supplement: Supplementary file 4 — Supplementary Information 4. [file 41598_2022_15021_MOESM4_ESM.pdf]
