# Supplementary material for: Estimating female malaria mosquito age by quantifying Y-linked genes in stored male spermatozoa
Source: Sci Rep. 2022 Jun 22;12:10570. doi: 10.1038/s41598-022-15021-z (PMC9217924; doi:10.1038/s41598-022-15021-z)
Supplement: Supplementary file 5 — Supplementary Information 5. [file 41598_2022_15021_MOESM5_ESM.docx]

**Supplemental Table 1. Primers and probes used in multiplexed qPCR method.** Final concentrations for each component used in qPCR are indicated in parentheses.

| **Target** | **Forward Primer (μM)** | **Reverse Primer (μM)** | **Hydrolysis Probe (μM)** |
| --- | --- | --- | --- |
| *GUY1* | TATGTAACGTACTCTATGAAGC (2) | GATCCGTTAAAAATTGACACC (2) | FAM-AACGTGACAGACAATAGCATAAGACC-IBFQ (0.7) |
| *YG2* | AGCCTCTATCCTATATAGACTTTG (2) | ATTTGTGACTGAACATGTCGCT (2) | TexRd-TCTGTTTCGGCTATCAAACCCTCCGT-IBRQ (0.7) |
| *RPS6* | GTATCGTTTCCCGCTACG (0.2) | ACGTATCCCTTCCATTCC (0.2) | HEX-ACGACCACAAGCTGCGTCACTTCTAT-IBFQ (0.1) |
| *KLH* | CATCAATGATCTCACCCG (0.2) | ACAACAAGCATATCTGTCC (0.2) | Quasar705-TGCCCTCGCCCTGATGC-BHQ2 (0.1) |
