# Supplementary material for: Estimating female malaria mosquito age by quantifying Y-linked genes in stored male spermatozoa
Source: Sci Rep. 2022 Jun 22;12:10570. doi: 10.1038/s41598-022-15021-z (PMC9217924; doi:10.1038/s41598-022-15021-z)
Supplement: Supplementary file 6 — Supplementary Information 6. [file 41598_2022_15021_MOESM6_ESM.docx]

**Supplemental Figure Legends**

**Supplemental Figure 1. Efficiencies of multiplex qPCR with differing polymerase conditions.**

**Supplemental Figure 2. Y-linked gene copies normalized to controls.** Copy numbers of *GUY1* (A) and *YG2* (B) were normalized to *KLH* (left) and *RPS6* (right) from blood fed mosquitoes prepared using the manual method. Fits (solid lines) with 95% confidence intervals (shaded regions) from zero-inflated negative binomial GLM are displayed.

**Supplemental Figure 3. Y-linked gene copies decrease with ovipositions for mosquitoes prepared using semi high throughput sample preparation.** Copy numbers of *GUY1* (A), *YG2* (B), and *RPS6* (C) were measured in mosquitoes differing in age from blood-fed (top) and blood-denied (bottom) cohorts prepared using a semi-high throughput sample preparation. Fits (solid lines) with 95% confidence intervals (shaded regions) from zero-inflated negative binomial GLM are displayed. The percent of PCR positive measurements as a function of age for Y-linked sequences and linear regression fits (solid lines) with 95% confidence intervals (shaded regions) are displayed for blood-fed (D) and blood-denied (E) cohorts.

**Supplemental Figure 4. More aggressive sample digestion is associated with higher Y-linked copy quantification.** Young blood-denied female mosquitoes were subjected to various bead beating and proteinase K digestion times and subjected to qPCR analysis. Whole mosquitoes (W) or posterior portions (P) were used in reactions containing KAPA3G Plant DNA polymerase (KAPA3G) or AmpliTaq Fast DNA Polymerase (Taq).
